# Implementation and Evaluation of a Gait Training Assistant for the Use of Crutches: Usability Study

**DOI:** 10.2196/51898

**Published:** 2024-08-16

**Authors:** Milan Anton Wolf, Leon Sauerwald, Felix Kosmalla, Florian Daiber, Antonio Krüger, Stefan Landgraeber

**Affiliations:** 1 Department of Ortopedic Surgery Saarland University Medical Center Homburg Germany; 2 German Research Centre for Artificial Intelligence (DFKI) Saarbrücken Germany

**Keywords:** telerehabilitation, orthopedics, digital gait trainer, orthopedic, gait, movement, walk, walking, crutch, crutches, sensor, sensors, rehabilitation, usability, digital health, physiotherapy, physical therapy, telehealth, telemedicine, eHealth, virtual, locomotor, locomotion

## Abstract

**Background:**

Surgical procedures on the lower extremities often require weight-bearing on crutches as part of the rehabilitation process. Orthopedic elective procedures enable patients to learn the correct use of crutches in a controlled preoperative setting. Digital assistance systems can safely circumvent a shortage of skilled staff and any contact restrictions that may be necessary.

**Objective:**

The usability of a newly developed gait training assistant (GTA) for the use of crutches will be evaluated. An intervention group trained to use crutches by the digital trainer will be compared with a control group trained to use crutches conventionally by a physiotherapist.

**Methods:**

As part of the development and implementation of a novel GTA, 14 patients learned to walk with crutches by completing specific exercises while receiving live feedback. Their movements were detected by a depth sensor and evaluated in real time. Specific parameters (step length, synchronous movement, crutch angle, and crutch distance to the feet) were compared with a control group (n=14) trained to use crutches by physiotherapists. The intervention group was also assessed by a physiotherapist. At the end of the study, the patients completed questionnaires to evaluate the usability of the system (Brooke’s System Usability Scale score) and patient satisfaction.

**Results:**

All patients trained by the novel GTA were able to use crutches correctly. The intervention group showed significantly better values for crutch angle (mean –6.3°, SD 3.5° vs mean –12.4°, SD 4.5°; *P*<.001) and crutch position (mean 3.3, SD 5.1 cm vs mean –8.5, SD 4.9 cm; *P*=.02). Both groups reported that they felt confident in the use of crutches, were able to follow the instructions, and enjoyed the training. Even though the majority (12/14, 86%) preferred physical therapy over a purely digital approach, most participants enjoyed using the system (13/14, 93%) and were interested in trying out other digital assistants (11/14, 79%). The usability of the GTA was rated above average by the majority (9/14, 64%) of the patients.

**Conclusions:**

The newly designed GTA is a safe method of teaching the use of crutches and is statistically superior to training by a physiotherapist. Even if patients prefer interaction with a physiotherapist over a purely digital approach, digital devices provide a safe and motivating opportunity to learn the essential locomotor skills for rehabilitation.

## Introduction

Following accidents or surgical procedures, it is often crucial for the healing process to provide support to the affected body part. In the case of lower limb injuries or surgeries, this can be accomplished by crutches. In the case of elective surgery, patients can receive preoperative instruction in a controlled environment several days prior to the procedure. In Germany, this training is typically provided by specialized physiotherapists.

The use of digital assistive devices has already become ubiquitous in clinical settings and serves to mitigate the strain on hospital personnel, reduce costs, and enhance patient rehabilitation [1]. Despite these advantages, advanced technology applications are currently used in patient with orthopedic care only in the context of studies [2-4].

Walking on crutches is a clearly structured movement sequence. However, the teaching of this skill requires significant staff resources. The projected increase in the number of elective orthopedic surgeries in the future is exacerbating the already persistent shortage of specialized health care personnel [5], which has been further intensified by the COVID-19 pandemic and the associated restrictions on physical contact [6].

The current digital aids for learning to walk with crutches do not provide the comprehensive support that a physiotherapist can offer [7,8]. Some crutches equipped with sensors can monitor proper use, but there are currently no commercially available systems that provide simultaneous guidance and assessment, which puts a strain on the resources of physiotherapists.

This study aims to evaluate the efficacy of a custom-designed gait training assistant (GTA) in teaching the proper use of crutches. The primary objectives of this study are to determine (1) the capability of the digital assistant to impart proper crutch-walking skills using a 3-point gait, (2) patient receptiveness toward the digital walking assistant, and (3) the competence of the digital crutch-walking training assistant in comparison to a human physiotherapist.

## Methods

### Study Design

A total of 28 patients who underwent elective orthopedic surgery and needed postoperative weight-bearing (3-point gait) on the lower extremity were included. Patients who could not follow the instructions of the GTA, for instance, due to neurological diseases, were excluded.

The patients were randomly assigned to the intervention group (n=14) or control group (n=14). The intervention group learned the proper use of crutches with the GTA. The control group learned the use of crutches with the help of a physiotherapist. Baseline data (age and sex) were collected using a survey. In the intervention group, satisfaction was assessed by a questionnaire specially developed for the study. In addition, the usability of the GTA was evaluated using Brooke’s System Usability Scale (SUS) [9]. The GTA and a physiotherapist evaluated the ability of both groups to use the crutches correctly.

### Ethical Considerations

A prospective clinical trial was conducted in August 2022 after obtaining patient consent and institutional review board approval from the Saarland Medical Association (318/21). Data was anonymized and no compensation was provided to participants.

### Implementation of the GTA

**Figure 1:**
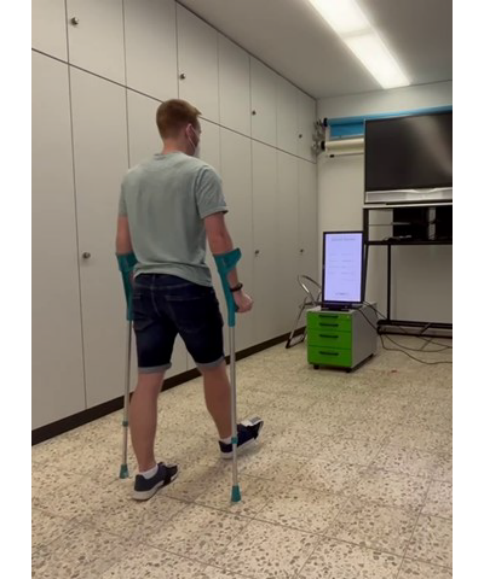
Training with the gait training assistant. The patient uses the crutches while the Kinect camera in front of him analyzes the crutches as well as the feet. The exercise to be completed is displayed with live feedback on the screen above the camera.

The GTA consists of a screen in portrait mode that gives visual guidance to the user and a 3D camera (Kinect v2 [Microsoft]) that tracks the position of the feet and crutches. While walking toward the screen, the gait pattern is automatically recognized and possible errors are brought to the attention of the user. The screen is also used to give instructions to the user (Figure 1). The user is guided through various exercises that aim to train a specific walking pattern (3-point gait). The software is designed so that each pattern can be described as a sequence of states, where each state specifies the crutch or the foot that needs to be moved. For 3-point gait, for instance, the user needs to move the injured leg, foot, or side and both crutches. Next, the noninjured leg, foot, or side is moved. This pattern is repeated. For simplicity, we focused on 3-point gait and forward movement only during this study, as other walking patterns and backward movement work similarly.

For simplicity, 2 infrared markers are attached to each crutch (handle and rubber foot) and each foot of the user to determine the movement of the crutches and feet. These reflective markers are extracted from the 2D infrared image and converted into 3D positions using the Kinect SDK. In this experiment, we focused on the 3-point gait. One step cycle was divided into smaller movements (eg, “Left foot and both crutches moving forward” and “Right foot moving forward”) that were derived by the 3D positions of the tracked reflective markers. Besides the necessary compound movements (foot and crutches), individual movements of crutches and feet were also recognized.

**Figure 2:**
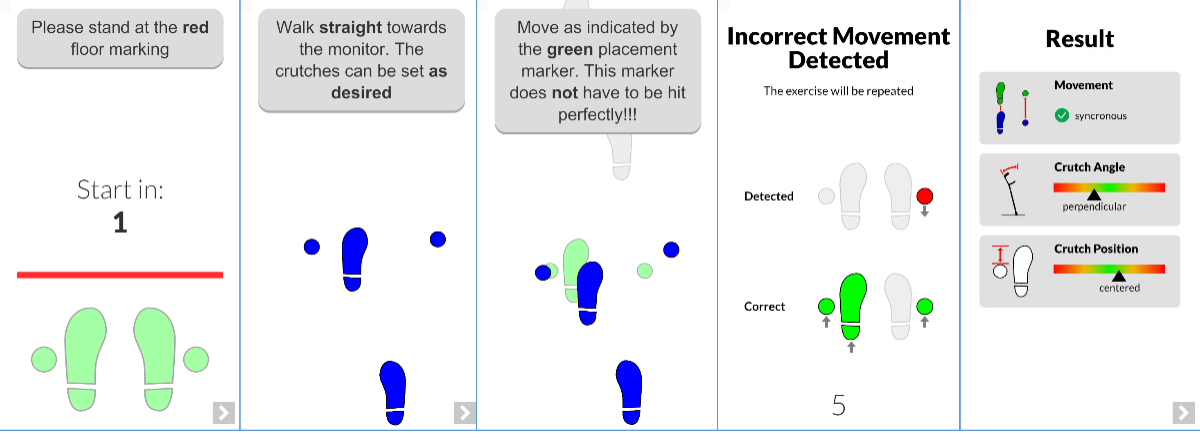
Excerpt from the instruction display with live feedback from gait training assistant. The first image (left) shows the start screen with the first instruction. The second image represents the instruction to use the crutches. The third image displays the live feedback. The fourth image signals an incorrect movement. The last image (right) provides the analysis of the exercise to the patient.

As the aim of the system is also to give corrective feedback during the training, the system tries to fit the currently detected movement to the gait pattern. If the system detects a wrong state or a wrong order of movements, it alerts the users and asks them to repeat the exercise (Figure 2).

The 3D positions of the tracked markers are also used to compute a set of metrics while the user is walking. These metrics include crutch angle, crutch position, and the synchronicity of the movement and help to provide feedback to the user after each exercise. The crutch angle describes the tilting of the crutch along the walking path toward the camera when it is placed on the ground. It is computed using the upper and lower markers of the crutch with some additional offset to match the tilting of the crutch cane. For the crutch position, the depth offset between the crutch and the foot is measured after they have moved. As the optimal position is the center of the foot, an offset of 15 cm is subtracted from the tracked foot position. To measure the synchronicity of the movement, the time when the crutch and foot are moving together is divided by the overall moving time for the current state.

### Statistical Analysis

The training performance of the GTA was assessed using an ANOVA. The number of correct runs during the analysis phase for each participant (independent variable) was compared between the 2 groups (dependent variable). The 2 groups were compared using a multivariate ANOVA (MANOVA), wherein 2 independent variables (crutch angle and crutch position) were simultaneously used to investigate the differences between the 2 groups. A correlation matrix was used to explore whether there was a correlation between the participants’ age and their perceived usability of the system.

## Results

### Baseline Data

In total, 28 participants were recruited for the primary study. The sample consisted of 13 female and 15 male participants with an age range of 18-73 (mean 41.1, SD 19.1) years. The participants’ height ranged from 1.55 m to 1.96 m. Approximately half (15/28, 54%) of the participants had prior experience with crutches, in most cases several years prior to the study. A total of 3 participants had used crutches in the last 12 months, although not for learning a 3-point gait (Table 1).

**Table 1:** Presentation of the baseline data of the patients.

Characteristics		Intervention group (n=14)	Control group (n=14)	Total (n=28)
**Sex, n (%)**				
	Female	6 (43)	7 (50)	13 (46)
	Male	8 (57)	7 (50)	15 (54)
Age (years), mean (SD)		44.4 (20.7)	37.8 (17.6)	41.1 (19.1)
Body height (cm), mean (SD)		173.1 (11.6)	175.3 (11.9)	174.2 (11.6)
**Used crutches before, n (%)**				
	Yes	6 (43)	9 (64)	15 (54)
	No	8 (57)	5 (36)	13 (46)

A total of 2 participants (P3 and P5; intervention group) could only partially perform the training with the GTA, as after a few exercises, the tracking function of 1 of the crutches was no longer shown in the live position visualization. Subsequent analysis was, therefore, not possible. However, they both filled out all the questionnaires. One other participant (P10; control group) had to be excluded from any analysis including the data recorded by the GTA, as no valid results were computed.

### Evaluation of the Training Performance by the GTA

**Figure 3:**
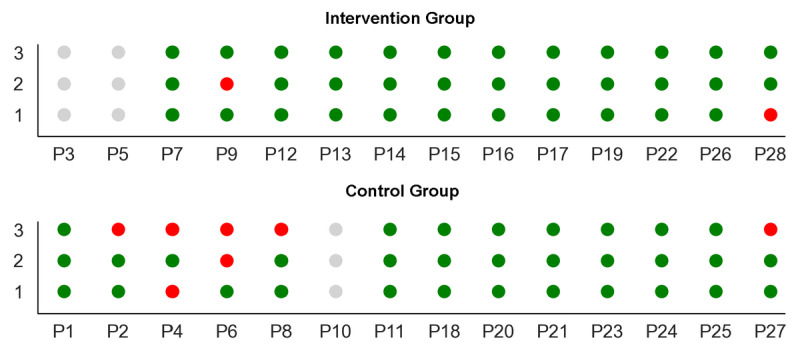
Status for each of the 3 exercises during the analysis: green: success; red: error (incorrect movement); and gray: excluded from the analysis. The upper image shows the results of the intervention group, and the lower image shows the results of the control group.

The GTA performance was determined by an analysis of the training sessions. Based on the number of incorrect exercises, the performance was extrapolated. Due to incomplete data collection, P3, P5 (intervention group), and P10 (control group) were excluded from the analysis. In the intervention group, 2 patients were found to have performed 1 of the 3 exercises incorrectly. In the control group, 3 patients performed 1 of the 3 exercises incorrectly and 2 participants performed 2 exercises incorrectly (Figure 3). The differences in the number of correctly performed exercises between the 2 groups were not statistically significant (*P*=.15).

The parameters selected for comparison of the 2 groups were crutch position and crutch angle. The reason for this is that step length and step speed are individual, patient-specific movements, and the physiotherapists, therefore, had to train synchronous movement depending on the patients’ physical condition.

The intervention group showed significantly better values for crutch angle (intervention group vs control group: mean –6.3°, SD 3.5° vs mean –12.4°, SD 4.5°; *P*<.001) and crutch position (intervention group vs control group: mean 3.3, SD 5.1 cm vs mean –8.5, SD 4.9 cm; *P*=.02). A MANOVA confirmed the superiority of the intervention group in the gait metrics (Table 2). Here, group assignment (intervention group and control group) was used as a factor, and crutch angle and crutch position were used as dependent variables. There was homogeneity of the error variances, as assessed by the Levene test (*P*>.05). The homogeneity of covariance was given by the Box test (*P*=.86). All tests revealed a statistical significance for *P*<.05.

**Table 2:** MANOVA^a^ analysis of gait metrics between the intervention group and control group^b^.

Test	Value	*F* test (*df*)	*P* value
Pillai trace	0.433	8.39 (2, 22)	.002
Wilks Lambda	0.567	8.39 (2, 22)	.002
Hotelling trace	0.763	8.39 (2, 22)	.002
Roy largest root	0.763	8.39 (2, 22)	.002

^a^MANOVA: multivariate ANOVA.

^b^The analysis showed that the training by the gait training assistant was significantly superior to the training by a physiotherapist.

### Evaluation of the GTA Training Performance by a Physiotherapist

The physiotherapist evaluated the ability to walk on crutches based on clinical experience using the parameters of step length, step speed, crutch angle, crutch position, and synchronous movement. Only the intervention group was evaluated, as an assessment of the control groups’ training performance by the physiotherapist was estimated to be too biased.

The analysis showed that almost all patients (10/12, 83%) trained on the GTA achieved a perfect result. Only 2 patients in the intervention group (P12 and P13) presented a slight deviation from the ideal score (Table 3).

**Table 3:** Gait training assistant performance of the intervention group, as evaluated by a physiotherapist. Synchronous movement is rated from 1 (not synchronous) to 5 (synchronous). All other metrics are rated from 1 to 5, with 3 as optimal value (eg, rating for step speed: 1=too slow, 3=optimal, and 5=too fast).

Parameters	Patient and rating											
	P7	P9	P12	P13	P14	P15	P16	P17	P19	P22	P26	P28
Step length	3	3	2	3	3	3	3	3	3	3	3	3
Step speed	3	3	2	3	3	3	3	3	3	3	3	3
Crutch angle	3	3	3	3	3	3	3	3	3	3	3	3
Crutch position	3	3	3	3	3	3	3	3	3	3	3	3
Synchronous movement	5	5	5	4	5	5	5	5	5	5	5	5

### Training Evaluation

**Figure 4:**
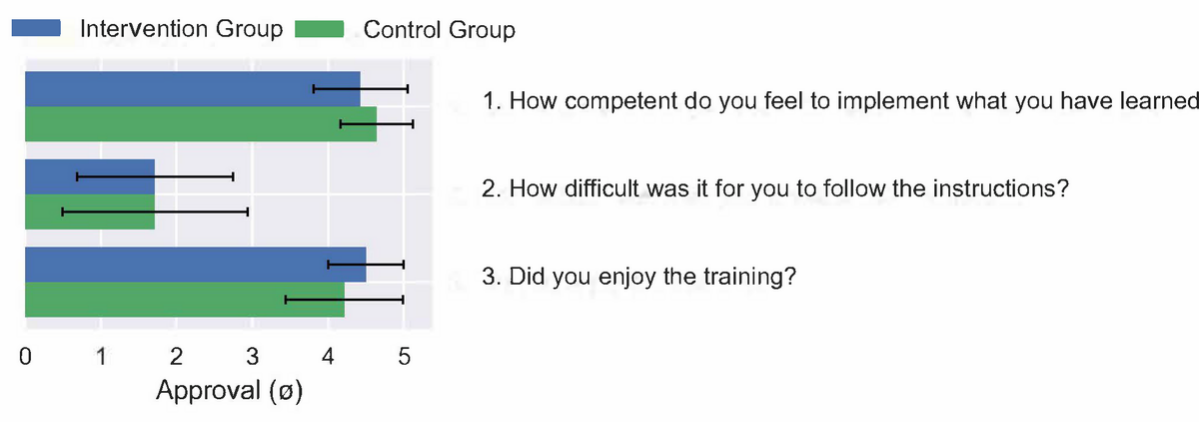
Evaluation of the training and comparison of patients from the control group and Intervention group. Rating from 1 (not at all) to 5 (very). Competence—intervention group: 4.43 (SD 0.62) and control group: 4.64 (SD 0.48). Difficulty—intervention group: 1.71 (SD 1.03) and control group: 1.71 (SD 1.22). Enjoyment—intervention group: 4.5 (SD 0.5) and control group: 4.21 (SD 0.77).

Both groups felt that they were competently trained (intervention group: mean 4.43, SD 0.62; control group: mean 4.64, SD 0.48; Figure 4). All patients reported that they were able to follow the instructions of the physiotherapists and the GTA (intervention group: mean 1.71, SD 1.03; control group: mean 1.71, SD 1.22). The participants in both groups indicated that they enjoyed the training, with the GTA performing slightly better (intervention group: mean 4.5, SD 0.5; control group: mean 4.21, SD 0.77). The differences between the groups were very small and were not statistically significant (MANOVA Wilks Lambda: *F*_3,42_=0.872; *P*=.47).

### Experience With the GTA

The participants in the intervention group were asked to complete a questionnaire with binary response options (yes or no). All participants indicated that they had learned to use the crutches safely and felt adequately prepared (14/14, 100% agreement), but 43% (6/14) of them indicated a desire for reevaluation by a physiotherapist. Nearly all participants (13/14, 93%) reported that they had understood the instructions provided by the GTA, 86% (12/14) reported that they were able to properly follow the instructions of the GTA, and 79% (11/14) stated that they would like to use a trainer for additional exercises. On the other hand, only 14% (2/14) of the participants wished to have purely digital rehabilitation without the support of a physiotherapist, while 29% (4/14) preferred training with a physiotherapist. Although 93% (13/14) of the participants enjoyed the gamified aspect of the GTA, only 14% (2/14) reported that it was more motivating than working with a physiotherapist (Table 4).

**Table 4:** Results of the questionnaire given to the patients in the intervention group. The results present the percentage of subjects who agreed with the question (answer: yes).

Question	Approval (n=14), n (%)
1. Have you learned how to safely use forearm crutches?	14 (100)
2. Do you feel prepared using forearm crutches confidently?	14 (100)
3. Would you like to be rechecked by a physical therapist?	6 (43)
4. Did you understand all the instructions of the program?	13 (93)
5. Have you been able to follow all the instructions of the program?	12 (86)
6. Would you like to learn more exercise through an interactive training program?	11 (79)
7. Would you perform a purely digital rehabilitation (ie, without a human physical therapist)?	2 (14)
8. Would you have preferred to learn how to use forearm crutches from a physical therapist?	4 (29)
9. Do you think the interactive trainer is more motivating than a physical therapist?	2 (14)
10. Do you like the playful concept of the interactive trainer?	13 (93)

### Assessment of GTA Usability

The usability of the GTA was evaluated using the established SUS [10]. A value of more than 68 can be considered above-average usability. A total of 9 (64%) of the 14 patients rated the usability of the GTA as above average. There was a tendency for younger patients to rate usability higher, but no significance was shown for this observation (Pearson *r*=–0.381; *P*=.18).

## Discussion

### Principal Findings

In the context of this study, a specifically designed GTA was used to teach patients how to use crutches. An RGB-D camera captured the position of the crutches, as well as the position of the study participant in the room. The training program guided the patients through a series of exercises and monitored their progress in real time, providing immediate feedback. This allowed simultaneous training and supervision. The results showed that compared with a control group that received conventional physiotherapy-led crutch training, the intervention group was not inferior and was able to effectively learn proper crutch use by the end of the exercise period. Although patients in the intervention group expressed positive feedback regarding the GTA and perceived it as useful, some remained skeptical about the potential for solely digital rehabilitation. The GTA was rated as having above-average user-friendliness.

Digital interventions are now increasingly integrated into everyday clinical practice for musculoskeletal rehabilitation. In particular, telerehabilitation [11] and mobile health (mHealth) applications [1] are available and prescribed by responsible therapists. At present, purely digital applications that work autonomously and can guide the patient while at the same time recognizing and preventing faulty exercises are not widely available. The programs available to date are mostly used only for the purpose of clinical studies [12]. Digital applications are rarely used in prerehabilitation.

The camera used in this study is the Microsoft Kinect camera released in 2010. Compared with other systems that only use body-worn markers (eg, Vicon [Vicon Motion Systems Ltd] and OptiTrack [NaturalPoint Inc]) for tracking (and are considered the gold standard for accuracy), the Kinect system can achieve similar precision in motion detection [13,14]. The Kinect system has already been embedded in several studies and successfully applied in rehabilitation programs [15,16]. The camera makes it possible to analyze gait without great expense or complex analysis systems. However, the short detection range of the camera is a disadvantage that can only be improved by using other more cost-intensive systems.

The analysis of the correct use of crutches can also be achieved by using sensors in the crutches themselves in addition to external systems such as a camera. Here, acceleration can be measured in addition to pressure. However, the established crutches are used almost exclusively in the measurement of load limits at partial weight-bearing. Tsuda et al [8] tried to overcome the limited detection range of the Kinect system (approximately 4 m) by using crutches with sensors. The disadvantage here is that the freedom of movement gained requires a wired connection to a computer and thus again leads to a disproportionately higher constraint.

In addition to the correct use of the crutches in terms of the movement and positioning of the crutches in the room [17], weight-bearing is important in some cases. Another study investigated forearm crutches with a built-in weight sensor. By using such a system, additional data can be displayed for the patient in real time, and the rehabilitation process can be further improved [18].

According to the available studies, the use of digital training systems is becoming increasingly popular and seems to be effective and safe [7,19,20]. In our study, there were no significant differences between the control and intervention groups. Although this is not proof of equality considering the low number of incorrect exercises, it can be concluded that the assistant can successfully teach a 3-point gait.

The comparison of crutch angle and crutch position reveals that in both cases, the intervention group achieved lower values with statistical significance. In terms of stability, it can be argued that the crutch angle closer to a vertical orientation provides greater contact between the rubber feet and the ground, thus reducing the probability of sliding. Regarding the crutch position, a smaller distance to the center of the foot can have the same effect. From this perspective, training with the digital assistant outperforms training with a physiotherapist. However, it is impractical to compare these metrics regarding a single optimal value. A slightly more tilted crutch can provide almost the same degree of stability, for example, due to the deformation of the rubber foot. Thus, a whole range of optimal values should be considered. This goes hand in hand with the evaluation of metrics by the physiotherapists, who rated all participants with a perfect score, that is, regardless of whether the angle was –14° or –4°. In this regard, both groups performed equally, and we can conclude that the digital assistant can compete with a human physiotherapist.

According to the completed questionnaires, both training approaches (GTA and analog) were similarly acceptable to the participants. Although the majority rejected a purely digital-based rehabilitation, most of them would have preferred training with a physiotherapist and even liked the concept of the assistant. However, the disadvantage of the system mentioned most often was the absence of interpersonal communication. A possible approach in future projects would be the use of artificial intelligence for communication or the use of professionals to monitor performance. Even if this again requires the involvement of staff, in the long term, the use of human resources is significantly lower.

### Conclusions

The use of the newly designed GTA is a safe method of learning to use the crutches and is statistically superior to training by a physiotherapist. Even if patients prefer interaction with a physiotherapist over a purely digital approach, digital devices provide a safe and motivating opportunity to learn essential locomotor skills for rehabilitation. Even though the range of the trainers is currently limited, in the future, the use of more advanced hardware can provide a comprehensive physiotherapeutic experience.

## Abbreviations

GTA: gait training assistant
MANOVA: multivariate ANOVA
mHealth: mobile health
SUS: System Usability Scale
